# Risk factors for severe Meibomian gland atrophy in a young adult population: A cross-sectional study

**DOI:** 10.1371/journal.pone.0185603

**Published:** 2017-09-28

**Authors:** Thao N. Yeh, Meng C. Lin

**Affiliations:** 1 Clinical Research Center, School of Optometry, University of California Berkeley, Berkeley, CA, United States of America; 2 Vision Science Graduate Group, University of California Berkley, Berkeley, CA, United States of America; Save Sight Institute, AUSTRALIA

## Abstract

**Purpose:**

Assess potential risk factors for severe Meibomian gland atrophy (SMGA) in a young adult population.

**Methods:**

Cross-sectional study using medical history and ocular surface examination to evaluate relationships with study outcomes: SMGA, tear lipid layer (TLL) thickness, non-invasive (NITBUT) and fluorescein (FTBUT) tear breakup times, and symptoms using the Standard Patient Evaluation of Eye Dryness (SPEED) questionnaire.

**Results:**

One hundred one participants (101; 202 eyes; Age: mean±SD = 22.3±4.0 years) completed the study. Hormonal birth control (HBC) use was the only significant risk factor for SMGA (p = 0.028). Female HBC users had 4.8 times greater odds of having SMGA compared to female HBC non-users (p = 0.028), but the odds of having SMGA was similar between female HBC non-users and males (p = 0.885). Multivariable analysis suggested that the relationship between SMGA and TLL thickness was dependent on HBC use. Compared to female HBC non-users without SMGA, TLL thickness for HBC users was estimated to be 10 nm thinner if SMGA was absent (p = 0.007) and 21 nm thinner if SMGA was present (p<0.001). SMGA status had no significant impact on TLL thickness among female HBC non-users (p = 0.552). The effect of TLL thickness on FTBUT was small but significant (p = 0.026). TLL thickness was not significantly associated with NITBUT (p = 0.349). Neither FTBUT nor NITBUT was significantly associated with the SPEED score.

**Conclusion:**

HBC use may be associated with SMGA, supporting the hypothesis that SMGA could lead to thinner TLL. However, less evidence was present to support that thin TLL could lead to clinically detectable tear film instability and subsequently to increased ocular dryness symptoms. Further investigation with a larger sample size is warranted to confirm these findings.

## Introduction

Lipids secreted from Meibomian glands are considered the main component of the superficial lipid layer of the tear film that protects the aqueous phase from evaporation and stabilizes the tear film by lowering surface tension [1,2]. It has been suggested that when the Meibomian glands become atrophied, keratinized, obstructed, or otherwise compromised to yield reduced or altered meibum in Meibomian gland dysfunction (MGD), these changes can result in a less stable tear film leading to increased aqueous evaporation rate [3–5]. Despite signs and symptoms being poorly correlated, it is believed that tear film instability can lead to a vicious cycle of tear hyperosmolarity and inflammation, ultimately resulting in adverse symptoms [5].

Studies have reported that Meibomian gland atrophy was associated with thinner tear lipid layer [6–10]. Of these studies, only one reports significant relationships between shorter tear breakup time and either thinner tear lipid layers or increased symptoms[10]. These discrepancies do not provide convincing evidence that alterations in the oil glands will be reflected downstream in tear film stability or symptoms. Conflicting results could be due to differences in study population, sample size, or instrumentation. Furthermore, these studies did not control for potential confounders in their analyses [6–10]. Both endogenous factors, such as age and sex, as well as exogenous factors, such as medications (e.g., hormonal birth control (HBC), anti-allergy, and antidepressants) and contact lenses are believed to influence one or many of the abovementioned ocular surface parameters [5,11].

This cross-sectional study aimed to determine the risk factors in a young adult population for severe Meibomian gland atrophy by accounting for various endogenous (e.g., age, sex) and exogenous (e.g., tobacco, medications, contact lens use) factors. The secondary aim was to investigate the potential downstream impact of severe Meibomian gland atrophy by evaluating relationships between severe Meibomian gland atrophy and tear lipid layer thickness, between tear lipid layer thickness and tear breakup time, and between tear breakup time and ocular dryness symptoms, while controlling for potential confounders. The results may help to identify individuals who are at greater risk of having severe Meibomian gland atrophy and to elucidate the overall impact of severe Meibomian gland atrophy on other ocular surface parameters.

## Methods

### Subjects

This was a cross-sectional study conducted at the University of California, Berkeley (UCB), Clinical Research Center. Study participants, who included non-contact lens and contact lens users, were recruited from the UCB campus and surrounding community. Subjects were required to have no history of ocular surgery or any active ocular inflammation or infection. Contact lens users were required to discontinue wearing their lenses 24 hours prior to their scheduled visits, and all participants were asked not to apply eye makeup on the day of their appointments. Written informed consent was obtained from all study participants, and the study adhered to the tenets of the Declaration of Helsinki. The study protocol was approved by the UCB Committee for Protection of Human Subjects.

Sample size estimates were calculated with two formulas [12,13] for the dichotomous outcome and one [14] for the continuous outcomes, using previously published population estimates for the main outcome severe Meibomian gland atrophy [15,16], and for the secondary outcomes tear lipid layer thickness [16,17], non-invasive tear breakup time [17,18], fluorescein tear breakup time [17–19], and SPEED score [17,20]. The highest sample size estimate for the main outcome of severe Meibomian gland atrophy, derived from the formula by Peduzzi, et al. [12], and from population estimates of severe Meibomian gland atrophy by Napoli, et al. [15], was 55 subjects in order to detect 3:1 odds of having severe Meibomian gland atrophy with 5% two-sided level of significance and 80% statistical power. The highest estimate among all outcomes was for fluorescein tear breakup time using the formula from Charan, et al. [14] and population estimates from Yeh, et al. [18] suggesting a sample size of 101 subjects to detect a minimum of a five-second difference in tear breakup time with 5% two-sided level of significance and 80% statistical power.

### Measurements and procedures

Table 1 lists all study procedures administered in this study in the order in which they were conducted, including references if methods were previously published. After providing written informed consent, study participants completed the Standard Patient Evaluation of Eye Dryness (SPEED) questionnaire [21] and the health history form, which requested information pertaining to current use of tobacco products, eye drops, medications (allergy, HBC, anti-depressives), eye makeup, and contact lenses.

**Table 1:** Study procedures listed in order performed.

Order	Procedure	Equipment/Materials	Measurements
1	Ocular and Medical History	Ocular and Medical History Form	• Contact Lens History (Use, duration, frequency) • Current Medications (Yes/No)
2	Symptoms Assessment	Standard Patient Evaluation of Eye Dryness (SPEED) Questionnaire[21]	• Severity Score (0–12) • Frequency Score (0–12) • Total Score (0–24)
3	Tear Film Interferometry and Number of Partial & Complete Blinks	Lipiview (TearScience, Morrisville, NC, USA)	• Average Lipid Layer Thickness (nm) • Number of Partial Blinks • Total Number of Blinks
4	Manual Non-invasive Tear Breakup Time (NITBUT)	Medmont Corneal Topographer E300 (Medmont Pty Ltd; Australia)	Tear Breakup Time (sec)
5	Anatomical Assessment	• Penlight • SL 120 (Carl Zeiss Meditec, Germany)	• Lagophthalmos (Yes/No) • Palpebral Aperture Size (mm)
6	Tear Breakup Time with fluorescein (FTBUT)	BioGlo™ Strip, Unisol Non-Preserved Saline	Tear Breakup Time (sec)
7	Corneal Staining	BioGlo™ Strips, Unisol Non-Preserved Saline	Sjogren’s International Clinical Collaborative Alliance (SICCA): Overall Score (0–4)[22]
8	Meibomian Gland Expressibility	Korb Meibomian Gland Evaluator™ (TearScience®; North Carolina)	Total Quality Score (0–45) and Quantity Score (0–45) recorded for each upper and lower lids [17,23]
9	Conjunctival Staining	1% Lissamine Green / 2% Sodium fluorescein ophthalmic drops	SICCA Score (0–3) per quadrant [22]
10	Line of Marx	1% Lissamine Green / 2% Sodium fluorescein ophthalmic drops	Score (0–3) was marked for each upper and lower lids [24]
11	Lid Wiper Epitheliopathy	1% Lissamine Green / 2% Sodium fluorescein ophthalmic drops	• Length Score (0–3)[25] • Sagittal Width Score (0–3)[25]
12	Meibography	Oculus Keratograph 5M (Oculus, Inc.; Arlington, WA, USA)	• Meiboscore (0–3) [26] • No. of Total Glands • No. of Atrophied Glands • No. of Tortuous Glands

Next were clinical measurements, which were taken on both eyes, always starting with the right eye. The clinical tests were ordered from least to most invasive, aimed at minimizing the impact of each test on subsequent tests. Details that could not be provided in the table are discussed below.

Performed first were non-invasive procedures that did not involve lid manipulation or instillation of drops, which included tear lipid layer thickness, non-invasive tear breakup time, and slit lamp examination with white light. The Lipiview® instrument (TearScience®; North Carolina, USA) measured tear lipid layer thickness (1 ICU unit ~ 1 nanometer) by conducting an interferometric color assessment of the tear film based on specular reflection, and it also measured the number of partial and total blinks within the 20-second measurement period. Participants were instructed to fixate on a light target while blinking normally during the measurement period. Non-invasive tear breakup time (NITBUT; seconds) was measured subjectively by the investigator with a Placido-disc-based corneal topographer (Medmont E300; Medmont Pty Ltd; Australia) three times per eye, alternating between eyes.

**Fig 1:**
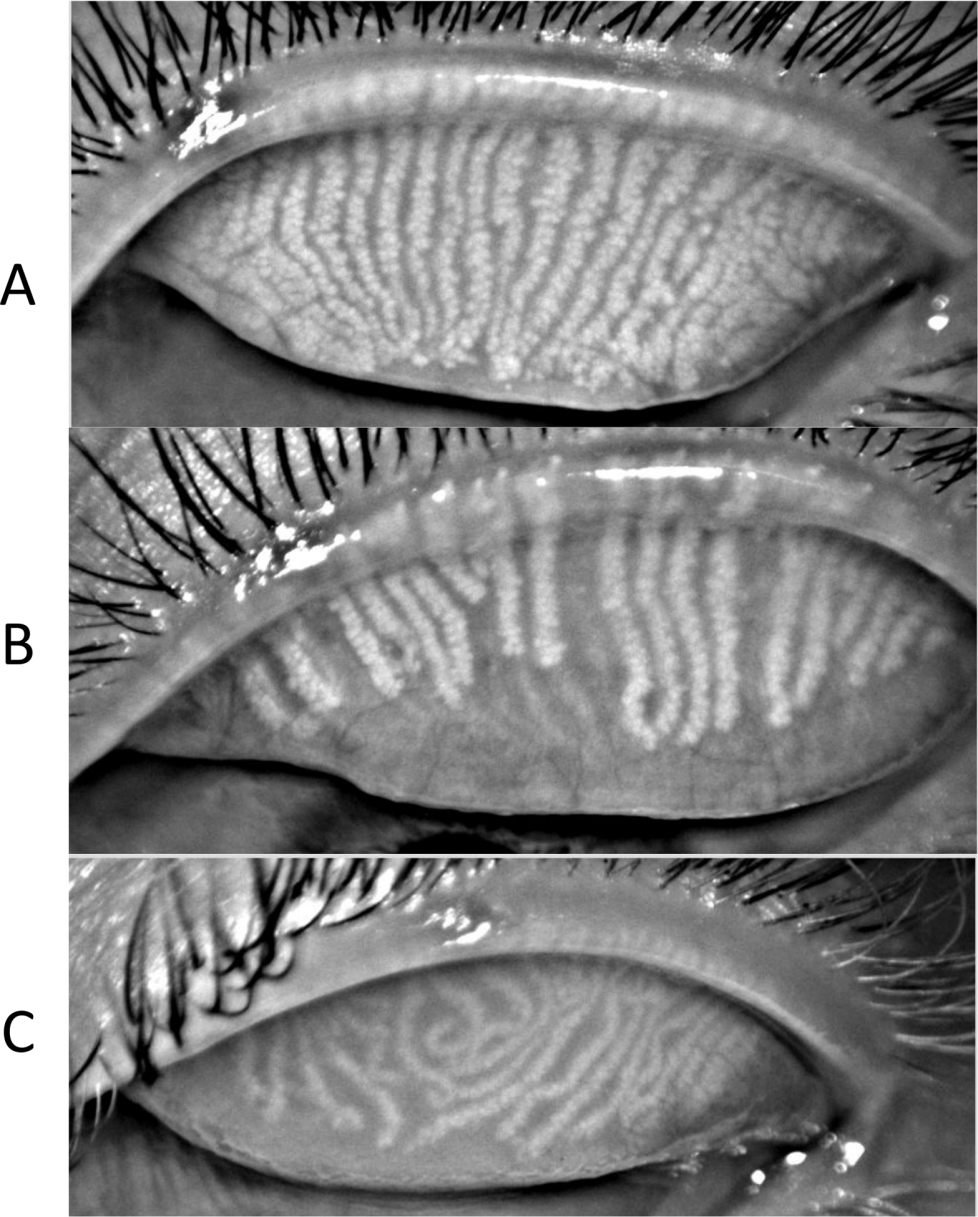
Meibomian gland characteristics. (A) Typical Meibomian gland orientation with glands running parallel and the full length of the eyelid, (B) Meibomian gland atrophy represented by shortened glands and trailing “empty” space, and (C) Meibomian gland tortuosity represented by glands with sharp bends (≥ 45°) or curly/hook appearances. Images courtesy of the Clinical Research Center, University of California, Berkeley.

Next, the invasive procedures were performed. Tear breakup time with sodium fluorescein (FTBUT; seconds) using BioGlo™ strips wetted with Unisol non-preserved saline were measured three times per eye, alternating between eyes. Corneal staining was scored immediately after FTBUT measurements on the SICCA scale [22]. Meibomian gland expression was then performed on the lateral, central, and medial regions of the lower and upper eyelid margins with a Korb Meibomian Gland Evaluator™. This procedure involved applying 10–15 seconds of gentle pressure on the skin inferior to the lower eyelid margin while the participant was in upgaze and superior to the upper eyelid margin while the participant was in downgaze. The secretions were scored based on quality and quantity [17,23]. Next, 1% lissamine green/2% fluorescein combination ophthalmic solution was instilled to assess conjunctival staining using the SICCA scale [22] and positioning of Marx’s line with respect to Meibomian gland orifices using the scale defined by Yamaguchi, et al. [24] Five minutes later, a second instillation of the lissamine green/fluorescein combination drop was instilled to assess length and width of lid wiper epitheliopathy after everting the upper eyelid using the scale previously defined by Korb, et al. [25] Finally, meibography images of both the upper and lower lids were scored based on the estimated percent area of Meibomian gland atrophy using Arita’s meiboscore scale [26] and evaluated for presence of tortuous Meibomian glands (bending ≥ 45°) and total number of visible Meibomian glands per eyelid (Fig 1).

### Statistical methods

The main outcome of interest was a binary variable representing presence/absence of severe Meibomian gland atrophy, where Meiboscore = 3 represents severe atrophy and Meiboscore<3 represents non-severe atrophy. The secondary outcomes were tear lipid layer thickness, tear breakup times, and SPEED score. Tear breakup times were transformed by natural logarithm to better approximate normality for statistical tests. Since both measures of tear breakup time (NITBUT and FTBUT) were moderately correlated (Pearson correlation = 0.53, p<0.001), multivariable models included only one of the measures at a time.

Analysis to examine direct, physiologically plausible relationships among the data collected was conducted using logistic regression for the binary outcome presence/absence of severe Meibomian gland atrophy, linear regression for the continuous outcomes tear lipid layer thickness and SPEED score, and log-linear regression for the log-transformed outcomes NITBUT and FTBUT. All regression models used the Huber-White standard error estimator clustered by subject (Stata/IC 14.0; vce(cluster) option) to account for within-subject correlations between eyes.

In the exploratory analysis, robust (clustered) regression models (1 dependent: 1 independent variable) were built for each direct, physiologically plausible relationship using the models as described above. Preliminary multivariable models (1 dependent: multiple independent variables) were then built for each outcome to include all significant independent variables from the respective exploratory analysis. Final models were selected by considering F-test p-values and testing assumptions using residual and other diagnostic plots and diagnostic tests, including Hosmer-Lemeshow.

Similar to the exploratory analysis, post-hoc comparison of ocular surface parameters between contact lens and non-contact lens users also used logistic regression for binary dependent variables and linear regression for continuous dependent variables, again, using the Huber-White standard error estimator clustered by subject (Stata/IC 14.0; vce(cluster) option) to account for within-subject correlations between eyes. In all of these models, the diagnostic tests were treated as dependent variables and contact lens use was treated as a binary independent variable.

## Results

One hundred one (101) subjects (202 eyes) between 18 and 41 years of age (mean±SD = 22.3±4.0) completed the study. Table 2 describes the population demographics and their reported use of various products that may interfere with ocular surface health, and Table 3 shows study population means for the study outcome variables.

**Table 2:** Demographics and product usage based on subject medical history [N = 101 (202 eyes)].

Characteristic	No. of Subjects
Sex	
*Female*	71
*Male*	30
Race	
*Asian*	55
*Non-Asian (White*, *Hispanics)*	29
*Other*	17
Contact Lens Status	
*Non-Users*	50
*Users*	51
Tobacco	
*Non-Users*	97
*Users*	4
Eye Drops	
*Non-Users*	79
*Users*	22
Allergy Medication	
*Non-Users*	98
*Users*	3
Hormonal Birth Control (HBC)	
*[Females only]*	
*Non-Users*	53
*Users*	18
Anti-Depression Medication	
*Non-Users*	98
*Users*	3
Make-Up Frequency	
*Never/Rarely*	61
*Frequently*	21
*Daily*	19

**Table 3:** Study distribution and means for main (severe Meibomian gland atrophy) and secondary outcomes. SD = Standard deviation, SPEED = Subjective Patient Evaluation of Eye Dryness.

	# of Eyes	Mean (SD)
**Any Severe Meibomian Gland Atrophy**		
**Not Present (Meiboscore<3)**	185	
**Present (Meiboscore = 3)**	17	
**Tear Lipid Layer Thickness (nm)**		59.7 (16.7)
**Non-invasive Tear Breakup Time (sec)**		10.71 (5.68)
**Fluorescein Tear Breakup Time (sec)**		5.90 (4.25)
**SPEED Score**		5.5 (4.1)

Table 4 is a summary of exploratory analysis for outcomes listed by columns and independent variables listed by rows. Relationships considered were ones in which the outcome and independent variable are believed to have a direct and physiologically plausible relationship. Intercepts, effect sizes, and P-values are listed for each relationship explored, with the significant relationships marked in bold, and relationships that were not examined for reasons described above are marked with a dash. Significant risk factors from exploratory analysis were included in the initial multivariable model for the respective outcome variable, and the final model was determined as described previously.

**Table 4:** Exploratory analysis results for each direct, physiologically plausible relationship between a potential risk factor (rows) and an outcome variable (columns).

Risk Factors	Outcomes														
	Severe MG Atrophy^A^			TLL Thickness^B^			ln(NITBUT)^C^			ln(FTBUT)^C^			SPEED^B^ Score		
	Int	Effect	P-value	Int	Effect	P-value	Int	Effect	P-value	Int	Effect	P-value	Int	Effect	P-value
Age	0.03	1.05	0.246	78.25	**-0.83**	**0.032**	2.24	-0.00	0.990	2.13	-0.02	0.061	3.19	0.11	0.369
Sex	0.11	0.48	0.384	59.27	1.45	0.664	2.23	0.01	0.922	1.54	0.13	0.326	6.00	-1.57	0.068
Race															
*Asian vs*. *Non-Asian*	0.08	1.47	0.587	59.39	2.75	0.447	2.15	**0.21**	**0.047**	1.51	0.17	0.217	5.45	-0.32	0.737
Contact Lens Years	0.05	**1.11**	**0.050**	63.18	**-0.84**	**0.005**	2.34	**-0.03**	**0.002**	1.71	**-0.03**	**0.001**	4.91	0.15	0.086
Tobacco Use	0.08	3.98	0.252	59.47	5.78	0.443	2.24	-0.18	0.634	1.58	0.04	0.868	5.60	-1.85	0.099
Eye Drop Use	0.08	1.56	0.544	61.79	**-9.61**	**0.002**	2.29	**-0.24**	**0.011**	1.62	-0.17	0.196	4.49	**4.78**	**<0.001**
Allergy Medication Use	0.08	6.03	0.158	60.32	**-20.82**	**<0.001**	2.24	0.01	0.984	1.58	-0.12	0.655	5.47	2.20	0.542
Anti-depression Medication Use	^+^	^+^	^+^	59.51	6.32	0.525	2.24	-0.07	0.773	1.58	-0.11	0.621	5.40	4.60	0.185
Hormonal Birth Control Category					**-**										
*FHBC*^*-*^ *vs*. *FHBC*	0.06	**4.76**	**0.028**	62.64	**13.30**	**<0.001**	2.25	-0.05	0.707	1.58	-0.15	0.403	5.81	0.74	0.496
*FHBC*^*-*^ *vs*. *Males*		0.88	0.885		-1.92	0.582		-0.00	0.990		0.09	0.492		-1.38	0.132
Make-Up Frequency															
*Never/Rare vs*. *Frequent*	0.06	2.22	0.320	59.26	0.76	0.851	2.22	0.17	0.119	1.53	0.16	0.196	5.59	0.22	0.832
*Never/Rare vs*. *Daily*		2.49	0.215		1.47	0.710		-0.11	0.320		0.08	0.618		-0.54	0.619
Meibomian Gland															—
*Any Severe Atrophy*	-	**-**	**-**	60.35	**-16.35**	**0.032**	-	-	-	-	-	-	-	-	-
*Any Tortuosity*	-	**-**	**-**	59.21	0.75	0.785	-	-	-	-	-	-	-	-	-
*Total Expressibility*	-	**-**	**-**	59.48	0.01	0.954	-	-	-	-	-	-	-	-	-
Line of Marx Position															
*Upper Eyelid*	-	-	-	59.88	-0.31	0.880	-	-	-	-	-	-	-	-	-
*Lower Eyelid*	-	-	-	58.93	0.82	0.650	-	-	-	-	-	-	-	-	-
Number of Blinks															
*Total*	-	-	-	62.86	-0.49	0.288	2.44	**-0.03**	**0.001**	1.72	-0.02	0.163	-	-	-
*All Partial*	-	-	-	56.50	**7.44**	**0.005**	2.11	**0.30**	**0.001**	1.40	**0.41**	**<0.001**	-	-	-
TLL Thickness	-	-	-	-	-	-	2.05	0.00	0.219	1.06	**0.01**	**0.007**	-	-	-
Lagophthalmos	-	-	-	-	-	-	2.21	0.22	0.094	1.56	0.18	0.168	-	-	-
Palpebral Aperture Size (mm)	-	-	-	-	-	**-**	1.52	**0.07**	**0.030**	1.51	0.01	0.854	-	-	-
Cornea SICCA Score	-	-	-	-	-	-	2.28	-0.04	0.355	1.63	-0.05	0.277	-	-	-
Conjunctival Total SICCA Score	-	-	-	-	-	**-**	2.34	**-0.08**	**<0.001**	1.62	-0.03	0.263	-	-	-
NITBUT (sec)	-	-	-	-	-	-	-	-	-	-	-	-	8.08	-1.14	0.098
FTBUT (sec)	-	-	-	-	-	-	-	-	-	-	-	-	6.32	-0.47	0.530
Lid Wiper															
*Length*	-	-	-	-	-	-	-	-	-	-	-	-	5.77	-0.28	0.306
*Sagittal Width*	-	-	-	-	-	-	-	-	-	-	-	-	5.83	-0.52	0.065

^A^ Robust logistic regression using Huber-White standard error estimator clustered by Subject ID; Odds ratio coefficient

^B^ Robust linear regression using Huber-White standard error estimator clustered by Subject ID

^C^ Robust log linear regression using Huber-White standard error estimator clustered by Subject ID

MG: Meibomian gland; TLL: Tear lipid layer; NITBUT: Non-invasive tear breakup time; FTBUT: Fluorescein tear breakup time; Int: Intercept; FHBC^-^: Females not using HBC; FHBC^+^: Females using HBC

^+^ No participants taking anti-depression medication had severe Meibomian gland atrophy

- Relationship not evaluated because indirect association or not physiologically plausible

**BOLD** values represent significant P-values

To allow for comparisons among males and both female groups (users and non-users of HBC), we generated a categorical variable called HBC Category, which included three groups: female HBC non-users, female HBC users, and males.

### Meibomian gland atrophy

In the study population, severe Meibomian gland atrophy was absent in 185 eyes and present in 17 eyes. Although both contact lens years and HBC Category were each significantly associated with Meibomian gland atrophy (Table 5), contact lens years was no longer significant (p = 0.080) in the multivariable model when both variables were included. As a result, the best model for severe Meibomian gland atrophy (Table 5) included only HBC Category as a risk factor. This model suggested that the odds of having severe Meibomian gland atrophy was 4.8 times greater for female HBC users than female HBC non-users (p = 0.028, 95% CI: [1.2, 19.1]) and that Meibomian gland atrophy severity was similar between female HBC non-users and males (p = 0.885, 95% CI: [0.2, 5.1]). Table 6 shows the distribution of eyes with severe Meibomian gland atrophy among the three HBC Category groups.

**Table 5:** Logistic regression model for severe Meibomian gland atrophy.

EFFECT	Severe Meibomian Gland Atrophy		
	Odds	P-value	95% CI
**Intercept**	0.06	<0.001	0.02, 0.15
**HBC Category**			
**(Control: Female Non-Users)**			
**Female Users**	4.76	0.028	1.19, 19.13
**Males**	0.88	0.885	0.15, 5.14

Confidence Interval (CI).

**Table 6:** Number of eyes with severe Meibomian gland atrophy (SMGA) among the hormonal birth control (HBC) groups.

	HBC Category			Total
	FHBC^-^	FHBC^+^	Males	
**SMGA Absent**	100	28	57	185
**SMGA Present**	6	8	3	17
**Total**	106	36	60	202

Female Non-Users of HBC (FHBC^-^); Female Users of HBC (FHBC^+^).

### Tear lipid layer thickness

**Fig 2:**
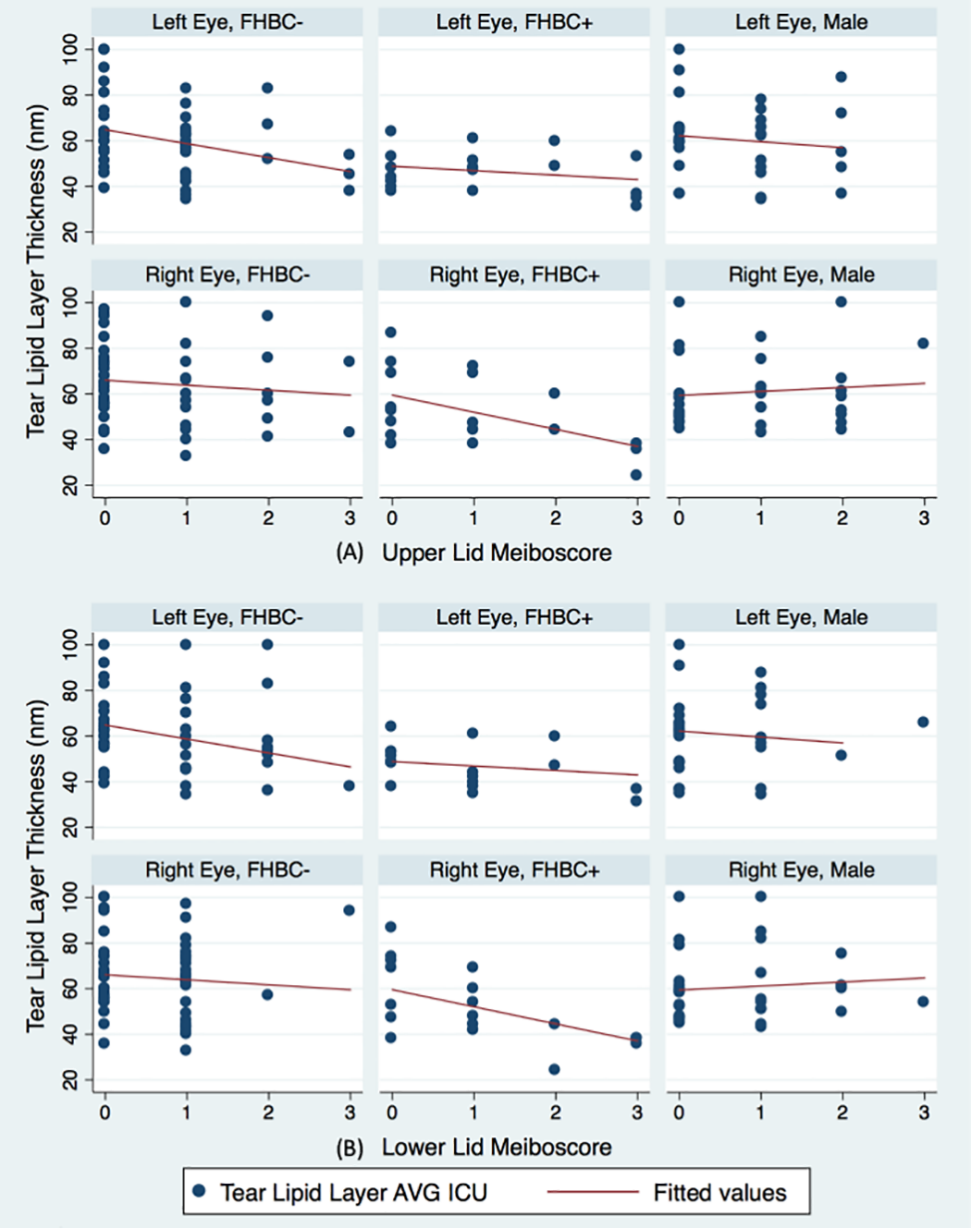
Tear film lipid layer thickness vs. Meiboscore, stratified by hormonal birth control use category. (A) Upper eyelid and (B) Lower eyelid. Meibomian gland atrophy severity based on Arita’s meiboscore [26] (0 = None (0% atrophy), 1 = Mild (up to 33%), 2 = Moderate (33–66%), 3 = Severe (>66%)). HBC: Hormonal birth control; FHBC^-^: Females not using HBC; FHBC^+^: Females using HBC.

With average tear lipid layer thickness measured from the LipiView® as the outcome, we found the best multivariable model was one that included years of contact lens use, partial blinking pattern, and the interaction between severe Meibomian gland atrophy and HBC Category (Table 7). The model suggested that tear lipid layer is 5 nm thinner for every 10 years of contact lens use (p = 0.032, 95% CI:[-1.0, -0.01]) and 6 nm thicker among all-partial blinkers compared to those who blink completely some or all the time (p = 0.033, 95% CI:[0.5, 10.5]). The model also suggests that female HBC users, regardless of their Meibomian gland atrophy status, had significantly thinner tear lipid layer than female HBC non-users who did not have severe Meibomian gland atrophy. Compared to female HBC non-users without severe Meibomian gland atrophy, tear lipid layer is thinner by approximately 10 nm for female HBC users without severe Meibomian gland atrophy (p = 0.007, 95% CI:[-16.5, -2.7]) and 21 nm thinner for female HBC users with severe Meibomian gland atrophy (p<0.001, 95% CI:[-28.0, -14.1]). In general, tear lipid layer thickness for males was similar to female HBC non-users, regardless of Meibomian gland atrophy severity. Fig 2 illustrates how tear lipid layer thickness varied with Meibomian gland atrophy severity across the three HBC groups.

**Table 7:** Linear regression model for tear lipid layer thickness.

EFFECT		Tear Lipid Layer Thickness		
		Estimate	P-value	95% CI
**Intercept**		62.9	<0.001	57.7, 68.0
**Contact Lens Years**		-0.5	0.032	-1.0, -0.01
**All Partial Blinks**		5.5	0.033	0.5, 10.5
**SMGA x HBC Category**				
**(Control: SMGA Absent, FHBC**^**-**^**)**				
**SMGA Absent**	**FHBC**^**+**^	-9.6	0.007	-16.5, -2.7
**SMGA Absent**	**Males**	-3.7	0.272	-10.3, 2.9
**SMGA Present**	**FHBC**^**-**^	-5.8	0.552	-25.1, 13.5
**SMGA Present**	**FHBC**^**+**^	-21.0	<0.001	-28.0, -14.1
**SMGA Present**	**Males**	5.5	0.524	-11.5, 22.4

Confidence Interval (CI); Hormonal Birth Control (HBC); Severe Meibomian Gland Atrophy (SMGA); Female Non-Users of HBC (FHBC^-^); Female Users of HBC (FHBC^+^).

### Tear film stability

#### FTBUT

The best multivariable model for ln(FTBUT) included tear lipid layer thickness and years of contact lens use (Table 8). Although shorter FTBUT was significantly associated with all or some complete blinking in the exploratory analysis, it is also considered a collider because it is an effect of both the exposure (tear lipid layer thickness) and outcome (FTBUT) and can alter their true relationship if included in the model. Therefore, blinking status was not included in the model. The final model estimates that FTBUT will increase by 7% for every 10 nm increase in tear lipid layer thickness (p = 0.026, 95% CI: [0.00, 0.01]) and decrease by 12% for every 5 years of contact lens use (p = 0.004, 95% CI: [-0.04, -0.01]). A tear lipid layer thickness change from 60 nm to 40 nm decreases FTBUT from 5.4 sec to 4.7 sec for non-contact lens users and from 4.8 sec to 4.2 sec for those with 5 years of contact lens wear, neither of which is clinically significant.

**Table 8:** Linear regression models for the log-transformed tear film stability measures.

	ln(FTBUT)			ln(NITBUT)		
EFFECT	Estimate	P-value	95% CI	Estimate	P-value	95% CI
**Intercept**	1.291	<0.001	0.904, 1.678	2.283	<0.001	1.966, 2.600
**Tear Lipid Layer Thickness (nm)**	0.007	0.026	0.001, 0.013	0.002	0.349	-0.003, 0.007
**Contact Lens Years**	-0.026	0.004	-0.043, -0.008	-0.021	0.015	-0.038, -0.004
**Conjunctival SICCA Score**	-	-	-	-0.073	0.002	-0.118, -0.027

Tear breakup time with fluorescein (FTBUT); non-invasive tear breakup time (NITBUT); Confidence Interval (CI).

#### NITBUT

As indicated in Table 4, NITBUT was significantly associated with several parameters in the univariate analysis but not with tear lipid layer thickness, which would be a more likely relationship. Without including blinking pattern for the same reason previously discussed, the best multivariable model suggested that shorter NITBUT on a long-transformed scale was not significantly associated with tear lipid layer thickness (p = 0.349) but that it was significantly associated with increased years of contact lens use (p = 0.015, 95% CI:[-0.038, -0.004]) and higher total conjunctival staining score (p = 0.002, 95% CI:[-0.118, -0.027]), which was largely driven by the nasal and temporal regions (Table 8). While a single unit change in conjunctival staining score resulted in less than a second change in NITBUT (clinically insignificant), a contact lens user of 10 years was estimated to have 2 seconds shorter NITBUT than a non-user.

#### Symptoms

Finally, of the potential risk factors for ocular dryness symptoms based on the SPEED score, univariable analysis revealed that symptoms were not significantly associated with FTBUT (p = 0.530) or NITBUT (p = 0.098). Instead, symptoms were strongly associated with eye drop use, such that the mean SPEED score was estimated to be 4.8 units higher for eye drop users than non-users (p<0.001).

### Post-hoc analysis: ocular surface differences between non-users and users of contact lenses

In relation to Meibomian gland atrophy, there was no significant difference between contact lens users and non-users when comparing the relative number of cases with severe Meibomian gland atrophy (p = 0.164) (Table 9). However, contact lens users were less likely to have any MG tortuosity in either the upper or lower eyelid (p = 0.031), and they had significantly shorter NITBUT (p = 0.009), reduced total Meibomian gland expressibility (p = 0.019), and higher SPEED scores (p = 0.002). Contact lens users had more partial (p = 0.012) and total blinks (p<0.001) per 20-second measurement period compared to non-users. There was no difference in tear lipid layer thickness or FTBUT between non-users and users of contact lenses.

**Table 9:** Comparison of diagnostic characteristics between non-contact lens and contact lens users.

	Non-Contact Lens	Contact Lens	P-value
	Mean(SD)		
Tear Lipid Layer Thickness (nm)	61(17)	55(16)	0.112^1^
Non-invasive Tear Breakup Time (sec)	11.8(5.7)	9.3(4.7)	**0.009**^1^
Fluorescein Tear Breakup Time (sec)	5.9(3.5)	5.6(5.1)	0.110^1^
Meibomian Gland Expressibility Total	40 (13)	34(14)	**0.019**^1^
SPEED Score	4 (3)	7 (4)	**0.002**^1^
Number of Partial Blinks	3.9 (2.8)	5.5 (4.0)	**0.011**^1^
Total Number of Blinks	5.0 (3.4)	7.9 (4.0)	**<0.001**^1^
	**Number of eyes**		
Severe Meibomian Gland Atrophy	5 / 95	12 / 90	0.164^2^
Present / Not Present			
Meibomian Gland Tortuosity	74 / 26	57 / 45	**0.031**^2^
Present/Not Present			
All Partial Blinks / Not All Partial Blinks	52 / 48	35 / 67	**0.033**^2^

^1^ Robust linear Regression with Huber-White standard error estimator clustered by Subject ID.

^2^ Robust logistic Regression with Huber-White standard error estimator clustered by Subject ID.

## Discussion

**Fig 3:**
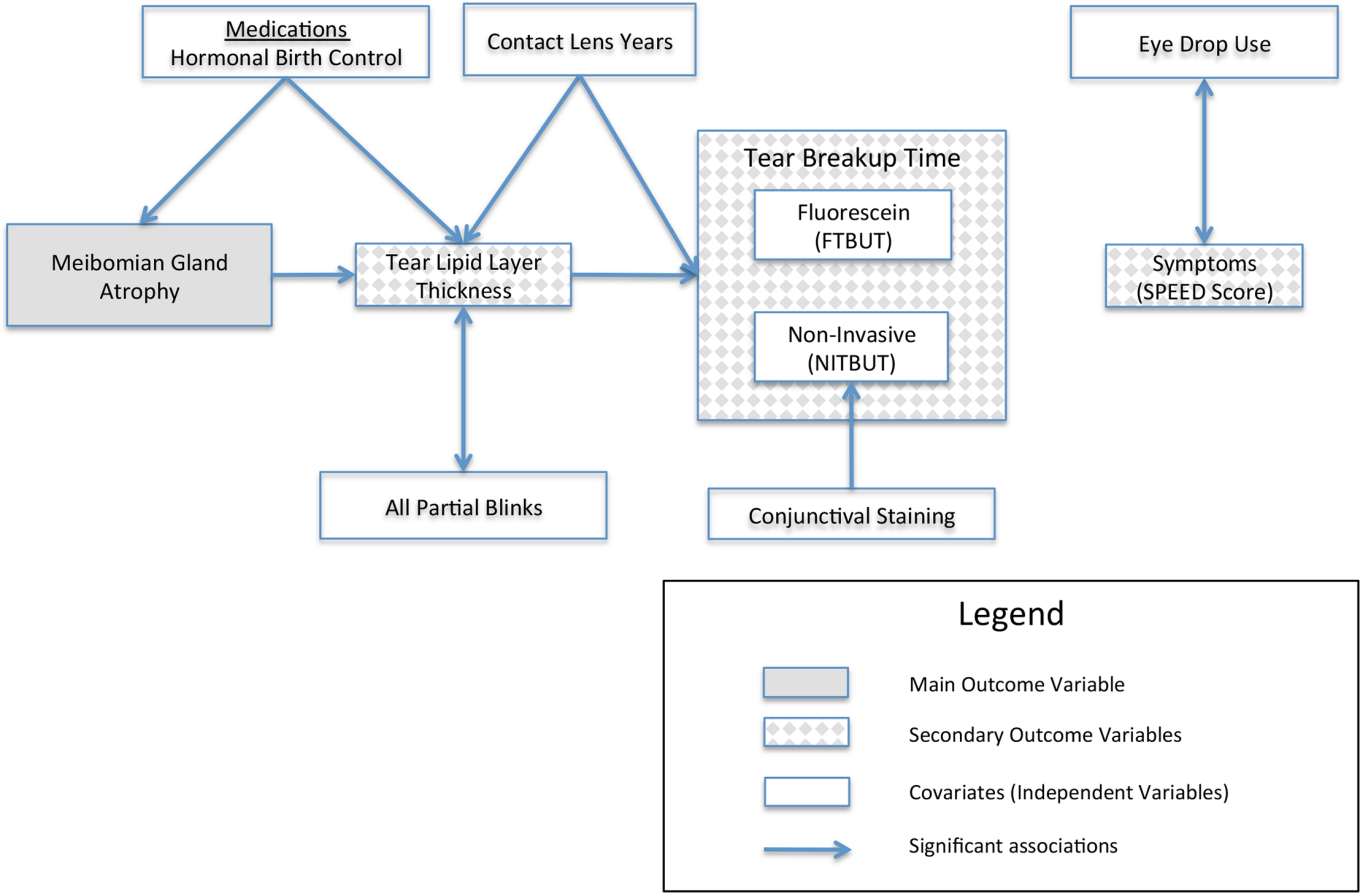
Summary of significant relationships based on final multivariable models between risk factors (arrow origin) and outcomes (arrowhead). SPEED = Subjective Patient Evaluation of Eye Dryness; NITBUT = Non-invasive Tear Breakup Time; FTBUT = Fluorescein Tear Breakup Time.

This cross-sectional study aimed to determine risk factors for severe Meibomian gland atrophy by accounting for both endogenous and exogenous factors and to investigate the potential downstream impact of severe Meibomian gland atrophy. Fig 3 summarizes the study findings. Of the potential risk factors considered for this young adult population, HBC use was the only significant risk factor for severe Meibomian gland atrophy. When we considered downstream effects, we found that severe Meibomian gland atrophy was associated with thinner tear lipid layer, shorter FTBUT was statistically but not clinically significantly associated with thinner tear lipid layer, and neither FTBUT nor NITBUT were associated with symptoms.

Since Meibomian glands are believed to be regulated by sex hormones [3,27,28], it is not surprising that HBCs can have a significant effect on Meibomian glands. HBCs act by decreasing androgen synthesis in the ovaries, adrenal glands, and peripheral tissues and reducing serum free testosterone levels by increasing sex hormone-binding globulin levels [29,30]. In the Meibomian glands, androgens appear to modulate lipid production and gene expression, while estrogens antagonize the actions of androgens by suppressing lipid synthesis [31,32]. While the relationship between HBC and Meibomian gland atrophy can be justified physiologically, other factors have also been linked to Meibomian gland atrophy. One is contact lens use, which is common among this young adult study population and has been shown, in some studies, to affect Meibomian gland atrophy [33,34]. Other studies, however, have found inconclusive or no evidence of such a relationship [19,35]. In the present study, duration of contact lens use alone was significantly associated with the presence of severe Meibomian gland atrophy, but it was no longer significant after controlling for HBC use. However, the small sample size of severe Meibomian gland atrophy does not allow proper analyses to determine the relative impact of contact lenses and HBC on Meibomian glands. Therefore, a larger, controlled study is warranted.

The relationship between severe Meibomian gland atrophy and thin tear lipid layer has been reported to be significant in other studies, despite differences in methodology [6–10]. In the present study, thinner tear lipid layer was significantly associated with greater years of contact lens use and complete blinking, but the relationships were not clinically significant. The use of HBC significantly affected the relationship between severe Meibomian gland atrophy and tear lipid layer thickness, such that females using HBC, regardless of Meibomian gland atrophy severity, had significantly thinner tear lipid layer than females not using HBC. To our knowledge, no study has reported the impact of HBC use on the relationship between Meibomian gland atrophy and tear lipid layer thickness.

The relationship between thinner tear lipid layer and shorter FTBUT was statistically significant, but the effect was too small to be detected clinically. NITBUT was not associated with tear lipid layer thickness. This relationship between tear-lipid thickness measured with the LipiView™ and tear film stability has been inconsistent in the literature [8,10]. It is unclear if the lack of association with tear film stability is due to poor instrument accuracy or precision or if tear-lipid thickness is an insufficient predictor of tear film stability. In general, we expect tear film stability to increase with increasing tear-lipid thickness, but there are a few examples that do not completely agree with this general impression. First, a previous study reported that tear-lipid stability could be maintained over a wide range of tear-lipid thicknesses and that instability would more likely occur below a certain threshold of tear lipid layer thickness [36]. Another example involves a tear-lipid layer that is thick, on average, but varies greatly over the measurement area [37]. It has been shown that a thick tear-lipid film can be associated with unstable tear film or a thin tear-lipid film can be associated with a stable tear film [37]. Therefore, understanding meibum quality (biophysical properties and/or composition) is just as important as lipid layer thickness when evaluating the impact of tear lipid layer on tear film stability [37–39].

This current study did not confirm an association between ocular dryness symptoms measured with the SPEED questionnaire and tear film stability, as hypothesized. This lack of association is consistent with a previous study that used similar methods, despite controlling for potential confounders [40]. Instead, we found that symptoms of ocular dryness were associated with eye drop use, which is likely a bidirectional relationship.

When evaluating the differences between users and non-users of contact lenses, we found that symptoms (SPEED), NITBUT, blink pattern, Meibomian gland expressibility and presence of tortuous Meibomian glands were significantly different between the groups. This study did not find a difference in presence of severe Meibomian gland atrophy between the two groups, which supports previously published findings by Machalinska, et al.[35], and Pucker, et al.[19], but contradict findings reported by Arita, et al.[26] and Alghamdi, et al.[34]. The discrepancy among these studies may be attributed to study population differences, such as demographics (age, race, and gender distributions), diet, and environmental factors. With many potential factors that may impact the ocular surface, a well-controlled, prospective study would help to elucidate the potential impact of contact lenses on the Meibomian glands.

In addition to the results related to the main and secondary outcomes, two other points are worth discussing. The first is the discrepancy between the two measures of tear film stability, NITBUT and FTBUT, which are highly correlated but yielded different results in the study models. This discrepancy may be attributed to the obvious differences in methods; the instillation of a fluorescein drop on the ocular surface to measure FTBUT can perturb the tear lipid layer, which in turn can cause a disruption in tear film stability that would not otherwise be seen with the Placido-based NITBUT measurement [41]. Although FTBUT is inherently variable due to the uncontrolled drop volume and concentration applied, the non-automated NITBUT measurements rely on visual observation of Placido rings that can be difficult to interpret, thus causing significant variability in the measurement.

The other result worth noting relates to blinking pattern. Subjects with all partial blinks during the measurement period had a thicker average tear lipid layer and better tear film stability (FTBUT and NITBUT). One explanation may be that eyes which have a thick tear lipid layer may have less of an urge to blink completely, and those with a thinner tear lipid layer are more inclined to blink completely in order to increase or restore tear lipid layer thickness. Ousler, et al. reported that there were slightly more partial blinks (52.9%) among normal eyes than among dry eyes (50.96%) and that total contact time (lid-to-lid) was seven times longer in dry-eye subjects than normal subjects (0.565 versus 0.080 seconds, respectively; P<0.001)[42]. While poor tear film can be the result of partial blinking tendencies in those individuals [43–46], we might consider that another group of individuals exist who are less inclined to blink completely due to the presence of a robust tear film. Another plausible explanation is that visual fixation on a light target in the LipiView® increases the tendency to blink partially. Several recent studies reported that reading and computer tasks increase the frequency of partial blinks [47–49], and it is possible that similar effects result with light fixation targets. It is unclear how such a task may impact those with healthier tear films differently, but if the general tendency is to blink partially and less frequently during these visual tasks, those with healthier tear films would likely tolerate those tendencies better than those with less robust tear films. Other possible reasons for discrepancies between the current study and previous findings on the relationship between blinking and tear lipid layer thickness may be attributed to differences in experimental setups, environmental conditions, and measurement algorithm (i.e., definition of partial blink vs. complete blink). While blinking results in this study were not captured under stringently controlled conditions, they are representative of data that would be collected by clinicians using the LipiView™ instrument on a healthy, young adult population.

This study was not without limitations. The young, healthy adult study population, limited the ability to assess age-related effects on the Meibomian glands reported by other studies [9,16,26]. While these results cannot necessarily be extended to the general population, they are useful for understanding the complex relationship that HBC has with the ocular surface in a population where its use is highly prevalent. Second, the main outcome of interest, severe Meibomian gland atrophy, was present in 17 of 202 eyes (8.4%) in this young population compared to 18% [15] and 43% [16] reported in older populations whose mean ages were 45 [15] and 57 [16] years, respectively. Although it is unclear if this current study accurately represents Meibomian gland atrophy distribution in the general population for this age group, these results are consistent with the expectation that the prevalence of severe Meibomian gland atrophy is higher in older age groups [9,16,26]. Third, this current study did not control for estrogen concentrations, type of progestin, diurnal or monthly hormonal variations, or the duration of HBC use, all of which may affect the relationships reported. Finally, the distribution of contact lens users among the female HBC groups was skewed and the sample size was small after stratification, making the analysis difficult to determine the independent effect of HBC and contact lens use on Meibomian gland structure and function.

In summary, HBC use may increase the odds for having severe Meibomian gland atrophy and affect the relationship between severe Meibomian gland atrophy and tear lipid layer thickness. Future studies with larger samples sizes are warranted to confirm these findings.

## Supporting information

S1 Dataset Complete dataset for study. (XLSX)

## References

[1] Holly FJ. Formation and rupture of the tear film. Exp Eye Res [Internet]. 1973 5 [cited 2017 Jul 6];15(5):515–25. Available from: http://linkinghub.elsevier.com/retrieve/pii/001448357390064X 4712544 10.1016/0014-4835(73)90064-x

[2] Tiffany JM. The Lipid Secretion of the Meibomian Glands. Adv Lipid Res [Internet]. 1987 [cited 2017 Jul 6];22:1–62. Available from: http://linkinghub.elsevier.com/retrieve/pii/B9780120249220500059 3328487 10.1016/b978-0-12-024922-0.50005-9

[3] Knop E, Knop N, Millar T, Obata H, Sullivan DA. The international workshop on meibomian gland dysfunction: report of the subcommittee on anatomy, physiology, and pathophysiology of the meibomian gland. Invest Ophthalmol Vis Sci [Internet]. 2011 3 [cited 2014 Feb 17];52(4):1938–78. Available from: http://www.pubmedcentral.nih.gov/articlerender.fcgi?artid=3072159&tool=pmcentrez&rendertype=abstract doi: 10.1167/iovs.10-6997c 21450915 10.1167/iovs.10-6997c PMC3072159

[4] King-Smith PE, Bailey MD, Braun RJ. Four Characteristics and a Model of an Effective Tear Film Lipid Layer (TFLL). Ocul Surf [Internet]. 2013 10 [cited 2017 Feb 1];11(4):236–45. Available from: http://www.ncbi.nlm.nih.gov/pubmed/24112227 doi: 10.1016/j.jtos.2013.05.003 24112227 10.1016/j.jtos.2013.05.003 PMC4313865

[5] Research in dry eye: report of the Research Subcommittee of the International Dry Eye WorkShop (2007). Ocul Surf [Internet]. 2007 4 [cited 2014 May 13];5(2):179–93. Available from: http://www.ncbi.nlm.nih.gov/pubmed/17508121 17508121 10.1016/s1542-0124(12)70086-1

[6] Pult H, Riede-Pult BH, Nichols JJ. Relation between upper and lower lids’ meibomian gland morphology, tear film, and dry eye. Optom Vis Sci [Internet]. 2012 3 [cited 2014 Jan 28];89(3):E310–5. Available from: http://www.ncbi.nlm.nih.gov/pubmed/22246333 doi: 10.1097/OPX.0b013e318244e487 22246333 10.1097/OPX.0b013e318244e487

[7] Matsumoto Y, Sato EA, Ibrahim OMA, Dogru M, Tsubota K. The application of in vivo laser confocal microscopy to the diagnosis and evaluation of meibomian gland dysfunction. Mol Vis [Internet]. 2008 1 [cited 2015 Feb 10];14:1263–71. Available from: http://www.pubmedcentral.nih.gov/articlerender.fcgi?artid=2447817&tool=pmcentrez&rendertype=abstract 18618006 PMC2447817

[8] Eom Y, Lee J-S, Kang S-Y, Kim HM, Song J-S. Correlation between quantitative measurements of tear film lipid layer thickness and meibomian gland loss in patients with obstructive meibomian gland dysfunction and normal controls. Am J Ophthalmol [Internet]. 2013 6 [cited 2014 Jan 28];155(6):1104–1110.e2. Available from: http://www.ncbi.nlm.nih.gov/pubmed/23465270 doi: 10.1016/j.ajo.2013.01.008 23465270 10.1016/j.ajo.2013.01.008

[9] Ban Y, Shimazaki-Den S, Tsubota K, Shimazaki J. Morphological evaluation of meibomian glands using noncontact infrared meibography. Ocul Surf [Internet]. 2013 1 [cited 2014 Jan 28];11(1):47–53. Available from: http://www.ncbi.nlm.nih.gov/pubmed/23321359 10.1016/j.jtos.2012.09.005 23321359

[10] Ji YW, Lee J, Lee H, Seo KY, Kim EK, Kim T. Automated Measurement of Tear Film Dynamics and Lipid Layer Thickness for Assessment of Non-Sjögren Dry Eye Syndrome With Meibomian Gland Dysfunction. Cornea [Internet]. 2017 2 [cited 2017 Jan 24];36(2):176–82. Available from: http://content.wkhealth.com/linkback/openurl?sid=WKPTLP:landingpage&an=00003226-201702000-00008 doi: 10.1097/ICO.0000000000001101 28060064 10.1097/ICO.0000000000001101

[11] Schaumberg DA, Nichols JJ, Papas EB, Tong L, Uchino M, Nichols KK. The international workshop on meibomian gland dysfunction: report of the subcommittee on the epidemiology of, and associated risk factors for, MGD. Invest Ophthalmol Vis Sci [Internet]. 2011 3 [cited 2014 Feb 18];52(4):1994–2005. Available from: http://www.pubmedcentral.nih.gov/articlerender.fcgi?artid=3072161&tool=pmcentrez&rendertype=abstract doi: 10.1167/iovs.10-6997e 21450917 10.1167/iovs.10-6997e PMC3072161

[12] Peduzzi P, Concato J, Kemper E, Holford TR, Feinstein AR. A simulation study of the number of events per variable in logistic regression analysis. J Clin Epidemiol [Internet]. 1996 12 [cited 2017 Jun 12];49(12):1373–9. Available from: http://www.ncbi.nlm.nih.gov/pubmed/8970487 8970487 10.1016/s0895-4356(96)00236-3

[13] Hsieh FY, Bloch DA, Larsen MD. A simple method of sample size calculation for linear and logistic regression. Stat Med [Internet]. 1998 7 30 [cited 2017 Jun 12];17(14):1623–34. Available from: http://www.ncbi.nlm.nih.gov/pubmed/9699234 9699234 10.1002/(sici)1097-0258(19980730)17:14<1623::aid-sim871>3.0.co;2-s

[14] Charan J, Biswas T. How to calculate sample size for different study designs in medical research? Indian J Psychol Med [Internet]. 2013 4 [cited 2017 Jul 3];35(2):121–6. Available from: http://www.ncbi.nlm.nih.gov/pubmed/24049221 doi: 10.4103/0253-7176.116232 24049221 10.4103/0253-7176.116232 PMC3775042

[15] Napoli PE, Coronella F, Satta GM, Iovino C, Sanna R, Fossarello M. A Simple Novel Technique of Infrared Meibography by Means of Spectral-Domain Optical Coherence Tomography: A Cross-Sectional Clinical Study. Paul F, editor. PLoS One [Internet]. 2016 10 31 [cited 2017 Jun 12];11(10):e0165558 Available from: http://dx.plos.org/10.1371/journal.pone.0165558 doi: 10.1371/journal.pone.0165558 27798696 10.1371/journal.pone.0165558 PMC5087862

[16] Finis D, Ackermann P, Pischel N, König C, Hayajneh J, Borrelli M, et al Evaluation of Meibomian Gland Dysfunction and Local Distribution of Meibomian Gland Atrophy by Non-contact Infrared Meibography. Curr Eye Res [Internet]. 2015 10 3 [cited 2017 Jun 12];40(10):982–9. Available from: http://www.ncbi.nlm.nih.gov/pubmed/25330304 doi: 10.3109/02713683.2014.971929 25330304 10.3109/02713683.2014.971929

[17] Satjawatcharaphong P, Ge S, Lin MC. Clinical Outcomes Associated with Thermal Pulsation System Treatment. Optom Vis Sci [Internet]. 2015 9 [cited 2017 Jun 13];92(9):e334–41. Available from: http://content.wkhealth.com/linkback/openurl?sid=WKPTLP:landingpage&an=00006324-201509000-00030 doi: 10.1097/OPX.0000000000000670 26192152 10.1097/OPX.0000000000000670

[18] Yeh TN, Graham AD, Lin MC. Relationships among Tear Film Stability, Osmolarity, and Dryness Symptoms. Optom Vis Sci [Internet]. 2015 9 [cited 2017 Jun 12];92(9):e264–72. Available from: http://content.wkhealth.com/linkback/openurl?sid=WKPTLP:landingpage&an=00006324-201509000-00020 doi: 10.1097/OPX.0000000000000649 26154693 10.1097/OPX.0000000000000649 PMC4924532

[19] Pucker AD, Jones-Jordan LA, Li W, Kwan JT, Lin MC, Sickenberger W, et al Associations with Meibomian Gland Atrophy in Daily Contact Lens Wearers. Optom Vis Sci [Internet]. 2015 9 [cited 2017 Jun 12];92(9):e206–13. Available from: http://content.wkhealth.com/linkback/openurl?sid=WKPTLP:landingpage&an=00006324-201509000-00012 doi: 10.1097/OPX.0000000000000650 26154690 10.1097/OPX.0000000000000650

[20] Asiedu K, Kyei S, Mensah SN, Ocansey S, Abu LS, Kyere EA. Ocular Surface Disease Index (OSDI) Versus the Standard Patient Evaluation of Eye Dryness (SPEED). Cornea [Internet]. 2016 2 [cited 2017 Jun 12];35(2):175–80. Available from: http://www.ncbi.nlm.nih.gov/pubmed/26655485 doi: 10.1097/ICO.0000000000000712 26655485 10.1097/ICO.0000000000000712

[21] Korb DR, Herman JP, Greiner J V, Scaffidi RC, Finnemore VM, Exford JM, et al Lid wiper epitheliopathy and dry eye symptoms. Eye Contact Lens [Internet]. 2005 1 [cited 2012 Oct 2];31(1):2–8. Available from: http://www.ncbi.nlm.nih.gov/pubmed/15665665 15665665 10.1097/01.icl.0000140910.03095.fa

[22] Whitcher JP, Shiboski CH, Shiboski SC, Heidenreich AM, Kitagawa K, Zhang S, et al A simplified quantitative method for assessing keratoconjunctivitis sicca from the Sjögren’s Syndrome International Registry. Am J Ophthalmol [Internet]. 2010 3 [cited 2014 Aug 11];149(3):405–15. Available from: http://www.pubmedcentral.nih.gov/articlerender.fcgi?artid=3459675&tool=pmcentrez&rendertype=abstract doi: 10.1016/j.ajo.2009.09.013 20035924 10.1016/j.ajo.2009.09.013 PMC3459675

[23] Greiner J V. A single LipiFlow® Thermal Pulsation System treatment improves meibomian gland function and reduces dry eye symptoms for 9 months. Curr Eye Res [Internet]. 2012 4 [cited 2014 Jan 28];37(4):272–8. Available from: http://www.ncbi.nlm.nih.gov/pubmed/22324772 doi: 10.3109/02713683.2011.631721 22324772 10.3109/02713683.2011.631721

[24] Yamaguchi M, Kutsuna M, Uno T, Zheng X, Kodama T, Ohashi Y. Marx line: fluorescein staining line on the inner lid as indicator of meibomian gland function. Am J Ophthalmol [Internet]. 2006 4 [cited 2014 Jun 10];141(4):669–75. Available from: http://www.ncbi.nlm.nih.gov/pubmed/16564801 doi: 10.1016/j.ajo.2005.11.004 16564801 10.1016/j.ajo.2005.11.004

[25] Korb DR, Herman JP, Blackie CA, Scaffidi RC, Greiner J V, Exford JM, et al Prevalence of lid wiper epitheliopathy in subjects with dry eye signs and symptoms. Cornea [Internet]. 2010 4 [cited 2014 Jun 10];29(4):377–83. Available from: http://www.ncbi.nlm.nih.gov/pubmed/20168216 doi: 10.1097/ICO.0b013e3181ba0cb2 20168216 10.1097/ICO.0b013e3181ba0cb2

[26] Arita R, Itoh K, Inoue K, Amano S. Noncontact infrared meibography to document age-related changes of the meibomian glands in a normal population. Ophthalmology [Internet]. 2008 5 [cited 2012 Sep 6];115(5):911–5. Available from: http://www.ncbi.nlm.nih.gov/pubmed/18452765 doi: 10.1016/j.ophtha.2007.06.031 18452765 10.1016/j.ophtha.2007.06.031

[27] Schirra F, Suzuki T, Richards SM, Jensen R V., Liu M, Lombardi MJ, et al Androgen Control of Gene Expression in the Mouse Meibomian Gland. Investig Opthalmology Vis Sci [Internet]. 2005 10 1 [cited 2017 Jul 14];46(10):3666 Available from: http://www.ncbi.nlm.nih.gov/pubmed/16186348 10.1167/iovs.05-0426 16186348

[28] Suzuki T, Schirra F, Richards SM, Jensen R V., Sullivan DA. Estrogen and Progesterone Control of Gene Expression in the Mouse Meibomian Gland. Investig Opthalmology Vis Sci [Internet]. 2008 5 1 [cited 2017 Jul 14];49(5):1797 Available from: http://www.ncbi.nlm.nih.gov/pubmed/18436814 10.1167/iovs.07-1458 18436814

[29] van der Vange N, Blankenstein MA, Kloosterboer HJ, Haspels AA, Thijssen JHH. Effects of seven low-dose combined oral contraceptives on sex hormone binding globulin, corticosteroid binding globulin, total and free testosterone. Contraception [Internet]. 1990 4 [cited 2017 Jul 14];41(4):345–52. Available from: http://linkinghub.elsevier.com/retrieve/pii/001078249090034S 2139843 10.1016/0010-7824(90)90034-s

[30] Wiegratz I, Kutschera E, Lee JH, Moore C, Mellinger U, Winkler UH, et al Effect of four different oral contraceptives on various sex hormones and serum-binding globulins. Contraception [Internet]. 2003 1 [cited 2017 Jul 14];67(1):25–32. Available from: http://linkinghub.elsevier.com/retrieve/pii/S0010782402004365 12521654 10.1016/s0010-7824(02)00436-5

[31] Sullivan DA, Jensen R V, Suzuki T, Richards SM. Do sex steroids exert sex-specific and/or opposite effects on gene expression in lacrimal and meibomian glands? Mol Vis [Internet]. 2009 1 [cited 2015 Oct 19];15:1553–72. Available from: http://www.pubmedcentral.nih.gov/articlerender.fcgi?artid=2728565&tool=pmcentrez&rendertype=abstract 19693291 PMC2728565

[32] Sullivan DA, Sullivan BD, Evans JE, Schirra F, Yamagami H, Liu M, et al Androgen deficiency, Meibomian gland dysfunction, and evaporative dry eye. Ann N Y Acad Sci [Internet]. 2002 6 [cited 2014 Feb 10];966:211–22. Available from: http://www.ncbi.nlm.nih.gov/pubmed/12114274 12114274 10.1111/j.1749-6632.2002.tb04217.x

[33] Arita R, Itoh K, Inoue K, Kuchiba A, Yamaguchi T, Amano S. Contact lens wear is associated with decrease of meibomian glands. Ophthalmology [Internet]. 2009 3 [cited 2012 Aug 24];116(3):379–84. Available from: http://www.ncbi.nlm.nih.gov/pubmed/19167077 doi: 10.1016/j.ophtha.2008.10.012 19167077 10.1016/j.ophtha.2008.10.012

[34] Alghamdi WM, Markoulli M, Holden BA, Papas EB. Impact of duration of contact lens wear on the structure and function of the meibomian glands. Ophthalmic Physiol Opt [Internet]. 2016 3 [cited 2017 Jul 6];36(2):120–31. Available from: doi: 10.1111/opo.12278 26890701 10.1111/opo.12278

[35] Machalińska A, Zakrzewska A, Adamek B, Safranow K, Wiszniewska B, Parafiniuk M, et al Comparison of Morphological and Functional Meibomian Gland Characteristics Between Daily Contact Lens Wearers and Nonwearers. Cornea [Internet]. 2015 9 [cited 2017 Jul 6];34(9):1098–104. Available from: http://content.wkhealth.com/linkback/openurl?sid=WKPTLP:landingpage&an=00003226-201509000-00019 doi: 10.1097/ICO.0000000000000511 26114822 10.1097/ICO.0000000000000511

[36] Lin MC, Svitova TF, Yeh TN, Yuen T, Zhou Y. Tear-lipid thickness vs. biophysical properties: which is more important for tear-film stability? In: American Academy of Optometry Annual Conference [Internet]. Denver, CO; 2014. Available from: http://www.aaopt.org/tear-lipid-thickness-vs-biophysical-properties-which-more-important-tear-film-stability

[37] Lin MC, Graham AD, Satjawatcharaphong P, Li W, Yeh TN, Lerma M, et al. Tear lipid layer thickness and variability both impact tear film stability [Internet]. Vol. 57, Investigative ophthalmology & visual science (ARVO Abstract). C.V. Mosby Co; 2016 [cited 2017 Jul 12]. Available from: http://iovs.arvojournals.org/article.aspx?articleid=2561685&resultClick=1

[38] Brown SI, Dervichian DG. The oils of the meibomian glands. Physical and surface characteristics. Arch Ophthalmol (Chicago, Ill 1960) [Internet]. 1969 Oct [cited 2017 Feb 1];82(4):537–40. Available from: http://www.ncbi.nlm.nih.gov/pubmed/5344949 10.1001/archopht.1969.00990020539019 5344949

[39] Herok GH, Mudgil P, Millar TJ. The effect of Meibomian lipids and tear proteins on evaporation rate under controlled in vitro conditions. Curr Eye Res [Internet]. 2009 7 [cited 2017 Feb 1];34(7):589–97. Available from: http://www.ncbi.nlm.nih.gov/pubmed/19899972 19899972 10.1080/02713680902972366

[40] Finis D, Pischel N, Schrader S, Geerling G. Evaluation of lipid layer thickness measurement of the tear film as a diagnostic tool for Meibomian gland dysfunction. Cornea [Internet]. 2013 12 [cited 2014 Jan 28];32(12):1549–53. Available from: http://www.ncbi.nlm.nih.gov/pubmed/24097185 doi: 10.1097/ICO.0b013e3182a7f3e1 24097185 10.1097/ICO.0b013e3182a7f3e1

[41] Greiner J V, Finnemore VM, Exford JM, Herman JP, Glonek T, Bueno EA, et al Effects of fluorescein instillation methods on the tear film lipid layer. Adv Exp Med Biol [Internet]. 2002 [cited 2016 Aug 7];506(Pt A):507–12. Available from: http://www.ncbi.nlm.nih.gov/pubmed/12613953 10.1007/978-1-4615-0717-8_71 12613953

[42] Ousler G, Abelson MB, Johnston PR, Rodriguez J, Lane K, Smith LM. Blink patterns and lid-contact times in dry-eye and normal subjects. Clin Ophthalmol [Internet]. 2014 5 [cited 2017 Aug 31];8:869 Available from: http://www.ncbi.nlm.nih.gov/pubmed/24833893 10.2147/OPTH.S56783 PMC4015796 24833893

[43] Kawashima M, Tsubota K. Tear lipid layer deficiency associated with incomplete blinking: a case report. BMC Ophthalmol [Internet]. 2013 1 [cited 2015 Oct 19];13:34 Available from: http://www.pubmedcentral.nih.gov/articlerender.fcgi?artid=3737109&tool=pmcentrez&rendertype=abstract doi: 10.1186/1471-2415-13-34 23855887 10.1186/1471-2415-13-34 PMC3737109

[44] Hirota M, Uozato H, Kawamorita T, Shibata Y, Yamamoto S. Effect of incomplete blinking on tear film stability. Optom Vis Sci [Internet]. 2013 7 [cited 2015 Oct 19];90(7):650–7. Available from: http://www.ncbi.nlm.nih.gov/pubmed/23770659 doi: 10.1097/OPX.0b013e31829962ec 23770659 10.1097/OPX.0b013e31829962ec

[45] Pult H, Riede-Pult BH, Murphy PJ. The Relation Between Blinking and Conjunctival Folds and Dry Eye Symptoms. Optom Vis Sci [Internet]. 2013 10 [cited 2015 Oct 19];90(10):1034–9. Available from: http://www.ncbi.nlm.nih.gov/pubmed/24067407 doi: 10.1097/OPX.0000000000000029 24067407 10.1097/OPX.0000000000000029

[46] Wan T, Jin X, Lin L, Xu Y, Zhao Y. Incomplete Blinking May Attribute to the Development of Meibomian Gland Dysfunction. Curr Eye Res [Internet]. 2015 8 19 [cited 2015 Oct 19];1–7. Available from: http://www.ncbi.nlm.nih.gov/pubmed/25835130 10.3109/02713683.2015.1007211 25835130

[47] Cardona G, García C, Serés C, Vilaseca M, Gispets J. Blink Rate, Blink Amplitude, and Tear Film Integrity during Dynamic Visual Display Terminal Tasks. Curr Eye Res [Internet]. 2011 3 28 [cited 2017 Sep 5];36(3):190–7. Available from: http://www.ncbi.nlm.nih.gov/pubmed/21275516 doi: 10.3109/02713683.2010.544442 21275516 10.3109/02713683.2010.544442

[48] Argilés M, Cardona G, Pérez-Cabré E, Rodríguez M. Blink Rate and Incomplete Blinks in Six Different Controlled Hard-Copy and Electronic Reading Conditions. Investig Opthalmology Vis Sci [Internet]. 2015 10 15 [cited 2017 Sep 5];56(11):6679 Available from: http://www.ncbi.nlm.nih.gov/pubmed/26517404 10.1167/iovs.15-16967 26517404

[49] Himebaugh NL, Begley CG, Bradley A, Wilkinson JA. Blinking and Tear Break-Up During Four Visual Tasks. Optom Vis Sci [Internet]. 2009 2 [cited 2017 Sep 5];86(2):E106–14. Available from: http://www.ncbi.nlm.nih.gov/pubmed/19156014 doi: 10.1097/OPX.0b013e318194e962 19156014 10.1097/OPX.0b013e318194e962

